# Oral Treatment with EGCG, Folic Acid, Vitamin B12, and Hyaluronic Acid Improves HPV Clearance and Counteracts Its Persistence: A Clinical Study

**DOI:** 10.3390/ijms26115251

**Published:** 2025-05-29

**Authors:** Giuseppina Porcaro, Maria Rosaria Pavone-Cossut, Sonia Moretti, Gabriele Bilotta, Cesare Aragona, Vittorio Unfer

**Affiliations:** 1 Department of Gynecology and Obstetrics, Women’s Health Centre, 05100 Terni, Italy; 2 National HIV/AIDS Research Center, Istituto Superiore di Sanità, 00161 Rome, Italy; 3 IVF Clinic, Alma Res Fertility Center, 00198 Rome, Italy; 4 Systems Biology Laboratory, Department of Experimental Medicine, Sapienza University, 00198 Rome, Italy; 5 Department of Gynecology and Obstetrics, UniCamillus—Saint Camillus International University of Health Sciences, 00131 Rome, Italy

**Keywords:** HPV, clearance, persistence, cervical lesions, EGCG, folic acid, vitamin B12, hyaluronic acid

## Abstract

Human papillomavirus (HPV) infection represents one of the most common sexually transmitted infections worldwide. However, the lack of effective therapeutic strategies to counteract viral infection and its persistence still makes the management of HPV a medical concern. Persistence is indeed a crucial issue in the context of HPV, as it may increase the risk of viral DNA integration into the host genome, thus exposing patients to tumoral progression. This clinical study aims to evaluate the effectiveness of a dietary supplement containing epigallocatechin gallate (EGCG), folic acid (FA), vitamin B12 (B12), and hyaluronic acid (HA) in improving HPV clearance and HPV-induced cervical lesions, and in counteracting viral persistence. A total of 106 patients who tested positive for HPV DNA were enrolled in this study and were treated daily for 6 months with a tablet containing EGCG (200 mg), FA (400 μg), B12 (1 mg), and HA (50 mg) (Pervistop^®^, Lo.Li. Pharma, Rome, Italy). A 6-month treatment with such combined molecules demonstrated a viral clearance in 85.8% of enrolled patients, while 92.3% of participants exhibited no more cervical lesions. Furthermore, 71.8% of patients with persistent infection tested negative to HPV DNA test after 6 months of treatment. The obtained data in this large population strongly support previous evidence on the efficacy of such molecules in the management of HPV infection by improving both viral clearance and related cervical lesions, and by targeting viral persistence.

## 1. Introduction

Human papillomavirus (HPV) contributes to a high global incidence of sexually transmitted infections (STIs), with over 80% of sexually active individuals expected to be exposed to HPV during their lifetime. Despite its high prevalence, counteracting HPV infection still remains a significant medical challenge on a global scale.

Among the concerns associated with HPV infection, the risk of promoting cancer represents a crucial aspect. According to their oncogenic potential, HPVs are classified into low-risk (LR) genotypes, which usually cause benign lesions (e.g., warts, condylomas, and recurrent respiratory papillomatosis) or precancerous lesions, and high-risk (HR) HPV genotypes, which are associated with the development of cancer of the lower genital tract, anus, and oropharynx both in men and women [1]. This classification is largely based on differences and similarities in the viral DNA sequence, particularly within the L1 open reading frame (ORF) gene region, where sequence similarity does not exceed 45% between subfamilies or 60% between genera. Among the most clinically relevant types, high-risk HPVs include HPV 16, 18, 26, 31, 33, 35, 39, 45, 51, 52, 53, 56, 58, 59, 66, 68, 73, and 82, while low-risk types include HPV 6, 11, 40, 42, 44, 54, 55, 61, 62, 71, 74, 81, 84, and 89 [2].

A critical stage in tumoral progression is represented by the integration of viral DNA into the host genome, which may occur in cases of persistent HPV infection [3]. In particular, the persistence of HR-HPV infection represents the primary etiological factor, leading to immune evasion mechanisms and contributing to the subsequent promotion of tumor development and progression. Indeed, about 90% of cervical and anal cancers are attributed to persistent HR-HPV infections [4].

As reported by Koshiol and colleagues, different definitions have been proposed for describing persistent HPV infections. In particular, most of the analyzed studies describe the minimum duration of HPV consecutive detection in a time frame between 6 and 12 months [5]. Several factors may contribute to the promotion of HPV persistence. For instance, female gender, HR-HPV infection, smoking, and co-infection with Ureaplasma spp. are just some of the factors associated with the persistence of genital HPV infection [6], reflecting the multifactorial etiopathogenesis of this condition. Although not all HPV genotypes integrate into the host genome, when integration occurs, the risk of developing cancer greatly increases. Viral DNA integration into the host genome may alter regular gene expression patterns, thus leading to chromosome instability [7]. In particular, once the viral genome is integrated, a breakpoint in the E2 viral genetic sequence occurs. As a consequence, this leads to the activation of the viral oncoproteins E6 and E7, which impact the activity of two main tumor suppressors, the transcription factor p53 and the retinoblastoma protein (RB) [8,9].

Preventing HPV infection and the potential associated risk of cancer is a crucial issue for public health. So far, vaccination and screening programs represent key public health strategies, with vaccination primarily aimed at preventing HPV infection, while screening focused on the early detection and management of pre-cancerous lesions before they progress into cancer. Providing a combined approach, ranging from vaccination and screening programs to the research of new therapeutic strategies, is a crucial topic considering that HPV infection and its persistence remain an unsolved medical problem.

Scientific research focused on finding novel therapeutic approaches to target the underlying mechanisms of infection to reduce viral load and improve patient care. Currently available treatments against HPV infection target only the clinical signs of infection, including condylomas or cervical lesions, but no specific therapies are able to counteract viral clearance and its persistence.

Recent evidence has reported promising results regarding the efficacy of some molecules in counteracting HPV infection, potentially addressing this therapeutic gap. Epigallocatechin gallate (EGCG) is a natural molecule with antiproliferative activity on cervical cancer cells by interfering with the HPV life cycle and suppressing the E6 and E7 oncoproteins, which are responsible for the viral oncogenic activity and cancer development [10,11]. In addition, EGCG may stimulate the Interferon (IFN) pathway, which is one of the escape mechanisms of HPV, thus reinforcing the innate anti-viral immunity against HPV [12].

Folic acid (FA) and vitamin B12 (B12) are two micronutrients whose plasmatic levels correlate with increased risk of cervical cancer and with the frequency of HR-HPV associated diagnosis: subjects with higher levels of folate and B12 are less likely (in 73% of cases) to test positive for HR-HPV types and more likely to test negative [13,14]. In addition, a consistent negative association between folate assumption and HPV infection was observed in a recent publication involving over 6700 HPV-positive women [15]. Moreover, low serum folate levels have been associated with an increased risk of CIN1 persistence and progression [16].

Transmission of HPV infection primarily occurs through sexual intercourse, commonly via skin–skin or skin–mucosal contact [17]; losing continuity in the epithelia represents the main strategy allowing the viral particles to penetrate and infect cells [18]. Therefore, molecules such as low- and very-low-weight molecular hyaluronic acid (LMWHA and vLMWHA), which promote the wound-healing process by stimulating the production of pro-inflammatory cytokines, play a crucial role in accelerating the re-epithelization process [19,20,21], thus preventing new reinfections.

Considering the large body of evidence supporting the activity of each of these molecules in the context of HPV [22], different preclinical and clinical studies have started to evaluate the beneficial effects of their combined use against HPV infection. Noteworthy, an in vitro study on HPV-positive cervical cancer cells (HeLa cells) highlighted for the first time the synergistic effect of EGCG, FA, B12, and HA in increasing apoptosis of tumoral cells by upregulating p53 and downregulating E6/E7 gene expression, respectively [23].

Recent clinical studies have started to explore the potential therapeutic effect of such molecules as oral administration for the recovery of HPV infection, reporting promising results in the resolution of the infection and cervical lesions. A pilot study by Aragona and colleagues highlighted that 17 out of 20 patients with HPV infection exhibited complete viral clearance and reported no cytological or histological evidence of lesions following the treatment [24].

In addition, a more recent clinical trial carried out by Tinelli and colleagues demonstrated that the use of an oral supplement containing EGCG, FA, B12, and HA reduced HPV positivity and improved related cervical lesions. In detail, a total of 86 patients orally treated with the combination of such molecules exhibited HPV clearance at 3 months as compared to the control untreated group [25]. In addition, cervical lesions improved after treatment, while in the control group, a small portion of patients developed HSIL cytology [25]. In addition to this, some case reports of patients with persistent HPV infection have supported the beneficial effects of this combination of molecules in reducing HPV presence and improving cervical-induced lesions [26].

The aim of this clinical study is to corroborate the effectiveness of the oral administration of EGCG, FA, B12, and HA in promoting HPV clearance and resolution of HPV-associated lesions, as well as counteracting HPV persistence in a larger number of patients.

## 2. Results

A total of 106 patients were enrolled and followed between August 2024 and February 2025, completing the 6-month intervention period. All enrolled patients completed the full 6-month treatment period, with no dropouts or missing data, and were included in the final analysis. Required inclusion criteria confirmed cervical infection, and all the patients analyzed presented a positive test result for HR-HPV. All patients’ baseline characteristic data, including age, presence of lesions, type of lesions, years of persistence, and anatomical site of infection, are summarized in Table 1. The effects of the 6-month treatment regimen consisting of EGCG, FA, B12, and HA were evaluated in terms of HPV clearance, clinical regression of related lesions, and clearance of persistent infections. Notably, none of the patients experienced any adverse effects during the treatment, suggesting good tolerability of the regimen.

### 2.1. Clearance of HPV After 6 Months of Treatment

**Figure 1:**
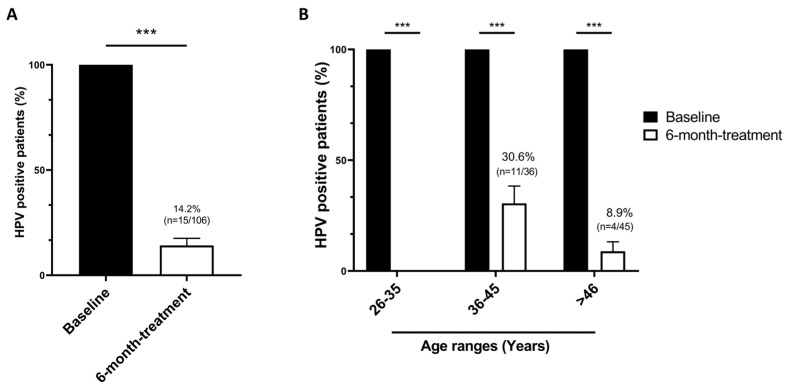
The effect of 6-month treatment with EGCG, FA, B12, and HA on HPV clearance. Graphical representation of the effect of EGCG, FA, B12, and HA on HPV clearance (**A**) and in different age ranges (**B**). At baseline (black columns), all patients tested positive for HPV DNA (100%). After the 6-month-treatment (white columns), HPV clearance statistically improved. Data are reported as means ± standard error of the mean. Statistical significance was evaluated using the Mann–Whitney U test for non-parametric comparison; *** *p* < 0.001.

At baseline, all women tested 100% positive for HPV. After 6 months of treatment, a significant HPV clearance (*p* < 0.001) was observed, with an 85.8% reduction (*n* = 91/106) in HPV positivity compared to baseline levels. Only 14.2% of patients (*n* = 15/106) were tested positive for HR-HPV genotypes (Figure 1A). To further explore the impact of such treatment across different age groups, we stratified patients into three age ranges (26–35, 36–45, and >46 years), ensuring a uniform distribution. Complete (100%; *n* = 25) and a nearly total (91.1%; *n* = 41/45) HPV clearance was observed at the extreme of the age spectrum, 26–35 and >46 years, respectively. Although the clearance rate was lower in the 36–45 age group (69.4%; *n* = 25/36), the results were statistically significant (*p* < 0.001) compared to baseline (Figure 1B).

### 2.2. Resolution of HPV-Induced Cervical Lesions After 6 Months of Treatment

**Figure 2:**
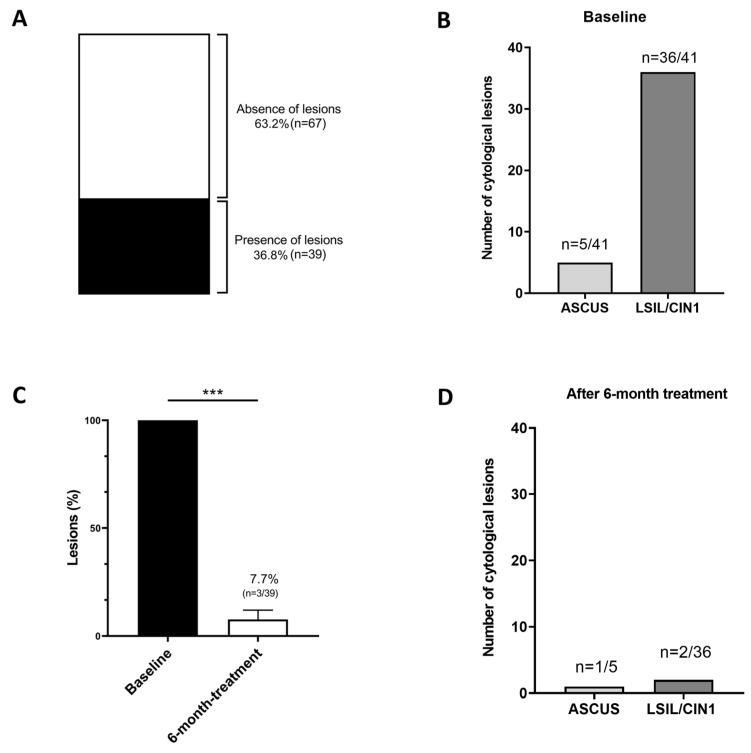
The effect of 6-month treatment with EGCG, FA, B12, and HA on HPV-induced cervical lesions. Baseline distribution of cervical lesions in HPV-positive patients undergoing a 6-month treatment regimen (**A**), and stratified by cytological classification (**B**). Graphical representation of the effect of EGCG, FA, B12, and HA on HPV-induced cervical lesions (**C**), then stratified by cytological classification (**D**). All patients included in the statistical analysis initially presented HPV-induced cervical lesions, with the baseline condition (black column) of lesion presence set at 100%. After 6 months of treatment (white column), 92.3% (36 out of 39 patients) showed resolution of HPV-induced lesions, while 7.7% (3 out of 39 patients) continued to exhibit cervical lesions (**C**). Data are reported as means ± standard error of the mean. Statistical significance was evaluated using the Mann–Whitney U test for non-parametric comparison; *** *p* < 0.001. Abbreviations: ASCUS (atypical squamous cells of undetermined significance); LSIL (low-grade squamous intraepithelial lesion); and CIN1 (cervical intraepithelial neoplasia grade 1).

At baseline, ThinPrep Papanicolaou Test (Pap test) analysis identified 46 HPV-induced cervical lesions among 39 patients (36.8%), while 67 patients (63.2%) showed no lesions (Figure 2A). The distribution of such lesions at baseline was as follows: LSIL/CIN1 (*n* = 36/41), ASCUS (*n* = 5/41) (Figure 2B). All patients with HSIL cytology (*n* = 5) had undergone surgery before the study began, so they were included only for HPV clearance assessment. After 6 months of treatment with the combination of EGCG, FA, B12, and HA, regression of cervical lesions was observed in 36 out of 39 patients (92.3%), while the remaining 3 out of 39 patients (7.7%) still exhibited cervical lesions (Figure 2C). These unresolved lesions included two lesions classified as LSIL/CIN1 and one as ASCUS, representing 20% (2 out of 36) and 5.5% (1 out of 5), respectively, of the total unresolved lesions compared to baseline (Figure 2D).

### 2.3. HPV Persistence After 6 Months of Treatment

**Figure 3:**
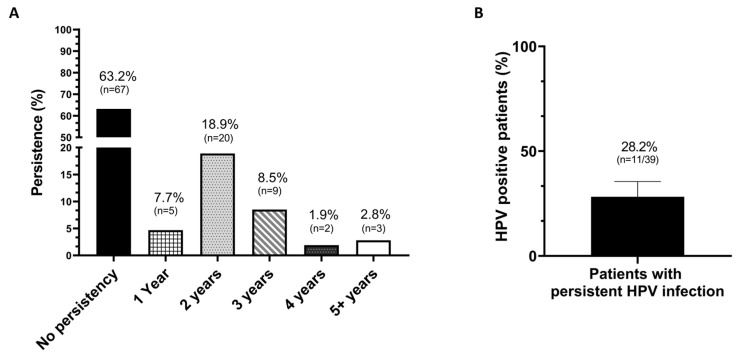
The effect of 6-month treatment with EGCG, FA, B12, and HA on persistent HPV infections. (**A**) Distribution of persistence of HPV infection according to the years of persistence indicated at baseline. (**B**) Percentage of patients with persistent infection who cleared the HPV infection.

Among the enrolled patients, 63.2% (*n* = 67) exhibited no persistent infection, while the remaining 36.8% (*n*= 39) exhibited HPV persistence, which is distributed as follows: 1 year (4.7%; *n* = 5), 2 years (18,9%; *n* = 20), 3 years (8.5%; *n* = 9), 4 years (1.9%; *n* = 2), and 5 or more years (2.8%; *n* = 3) (Figure 3A). By analyzing data according to HPV persistence, among the 39 patients with persistent infection for more than one year, 28 participants (71.8%) tested negative after the 6-month-treatment regime for any HPV genotypes, while 11 out of 39 patients (28.2%) remained positive on the HPV DNA test (Figure 3B).

## 3. Discussion

This open-label, single-arm clinical trial supports the effectiveness of oral administration of EGCG, FA, B12, and HA in counteracting HPV infection and its persistence, as well as improving related cytological lesions.

Several previous studies have evaluated the positive effects and efficacy of the individual molecules described in the context of HPV infection. In particular, EGCG has antiproliferative and proapoptotic activity, which mediates the activation of E6/E7, thus counteracting viral integration [12,27,28]; B12 and FA are methylation agents involved in blocking viral proliferation and persistence [29]; and HA may prevent HPV infection by restoring the integrity of the epithelium [21,30]. Notably, a recent in vitro study demonstrated for the first time the synergistic effect of these molecules in counteracting HPV infection by increasing apoptosis and p53 expression in HPV-infected cervical cells [23], thus paving the way for more comprehensive clinical evaluations.

The data obtained in this study are in line with previous clinical results, demonstrating that administration of this combination of molecules improved HPV clearance and cervical lesions, evaluated at both 3 months and 6 months [24,25,26]. As reported in our Supplementary Materials, preliminary ongoing results revealed a significant positive effect of the molecules on HPV clearance already after a 3-month treatment (Figure S1). However, given the limited number of patients, further analyses are needed to draw definitive conclusions at this specific time point.

In addition to HPV DNA testing, enrolled patients underwent Pap test analysis to evaluate cervical lesions. After 6-month treatment with the compound containing EGCG, FA, B12, and HA, almost all patients reported no lesions, with only 3 still presenting ASCUS or LSIL. This is a crucial result considering the management of cervical lesions in clinical practice and the lack of effective therapeutic strategies. Indeed, to date, clinical guidelines recommend only monitoring patients with low-grade lesions until they either spontaneously clear the infection or develop cervical neoplasia. On the other hand, in the case of high-grade lesions, surgery is the main strategy, even though it may not guarantee the complete clearance of the virus and/or the recurrence of new lesions [31]. In light of this, having molecules able to improve cervical lesions represents a great option to prevent tumoral progression. In addition, by targeting HPV clearance, such a combination of molecules represents a promising medical tool for improving clinical therapeutic outcomes in patients with HPV infection.

To date, vaccination and screening programs are the main options to prevent HPV infection. Indeed, the World Health Organization (WHO) recommends including HPV vaccines in national immunization programs, highlighting the importance of considering the prevention of cervical carcinoma and other HPV-associated diseases as a public health priority [32].

Notably, as reported in the Supplementary Materials Section, our ongoing preliminary data revealed a positive effect of EGCG, FA, B12, and HA supplementation on cervical lesions already after 3 months of treatment (Figure S2). However, as mentioned before, further analysis is needed to confirm these trends and draw definitive conclusions regarding the positive effect of these molecules at this specific timeframe.

Interestingly, the analysis of HPV clearance according to the age of the enrolled women revealed a statistically significant improvement across all age groups, although it was slightly less pronounced in the 36–45 age group. This probably correlates with the fact that the 36–45 range may represent a more challenging timeframe to counteract HPV infection, potentially due to factors such as age-related changes in immune response or prolonged exposure risks [33].

Considering the crucial role of persistence in HPV infection and tumoral progression, we focused on the subgroup of the study population presenting persistent HPV infection. Persistence is a crucial risk factor for tumoral progression as it may increase the possibility of viral DNA integration into the host genome, leading to the activation of E6/E7 proteins and the potential progression toward cancer. In light of the obtained results in counteracting HPV persistence after 6 months of treatment, the studied molecules become highly relevant in the management of the infection. Targeting HPV persistence can also contribute to reducing the risk of tumoral progression. These data are strongly in line with a previous study in which the authors demonstrated the positive effects of such molecules in improving viral persistence in 5 women with persistent HPV infection [26]. Furthermore, a previously published case report highlighted the effectiveness of this treatment even in a case of a 9-year HPV persistent infection, paving the way for further studies [34].

As above mentioned, clinical guidelines for managing HPV infection provide a “wait and see” approach relying on viral spontaneous regression. As reported in the Supplementary Materials Section (Figure S3), we compared our results with extrapolated spontaneous regression data from previously published articles [35,36,37,38], thus overcoming the lack of a control group in our investigation, which could represent a limitation to the observed findings. It is worth noting that these studies differ in terms of the number of involved patients, patients’ ethnicity, and age range, which may limit the applicability of these findings to current clinical contexts. Furthermore, such studies are outdated, relying on older diagnostic techniques that may not necessarily meet modern standards. Concomitant factors, including vaccination programs, changes in sexual behavior, and advancement in public health standards, may potentially influence the rate of spontaneous HPV clearance. Therefore, to address these limitations, we also compared our findings with extrapolated data from control patients in more recently published studies [24,25], which are more reliable since they employ more contemporary methodologies and reflect current population dynamics. The development of therapeutic options that may accelerate the HPV healing process represents a promising advancement in managing HPV clearance and its prevention. Considering the crucial role of immune markers in HPV persistence and progression—as reported in the recent publication by Guo et al. 2025 [39]—further studies should incorporate immune profiling and viral load monitoring to better understand the biological pathways influenced by the molecules in this dietary supplement. In addition, further clinical studies should also investigate differential treatment responses across HPV genotypes to better understand potential variations in viral clearance and lesion regression.

In conclusion, considering the high incidence of HPV infection and the lack of effective therapeutic strategies to counteract its persistence, the use of these combined molecules represents a crucial step forward in the daily care of HPV-positive patients.

## 4. Materials and Methods

### 4.1. Patients

This study is a single-arm, open-label clinical trial. Participants were followed from August 2024 to February 2025 according to standard clinical practice and in compliance with the Declaration of Helsinki. The study was approved by the Internal Review Board of Clinica ALMA RES (Approval No. 014/2022) and registered on ClinicalTrials.gov (Ref. No. NCT06661083). An informed consent document was signed by all participants before starting the study.

Women older than 18 years and positive for HPV DNA at baseline were enrolled in this study. Exclusion criteria were as follows: (i) ongoing pregnancy or breastfeeding, (ii) use of other products containing EGCG or green tea, and (iii) primary or pharmacologically induced immunodeficiency. The specific objective of this study was to evaluate whether the combination of EGCG, FA, B12, and HA improves HPV clearance and promotes cervical lesion regression. The primary outcome of this study was the clearance of HPV infection, evaluated as negative HPV DNA test results after 6 months-treatment. Secondary outcomes included the improvement or disappearance of cervical lesions and clearance of persistent HPV infection at the end of the oral treatment period.

### 4.2. Treatment Regimen

All enrolled patients followed an oral treatment regimen consisting of 1 tablet/day of a dietary supplement containing a combination of 4 molecules. Each tablet contained 200 mg of EGCG, 400 µg of FA, 50 mg of HA, and 1 mg of B12 (Pervistop^®^, Lo.Li Pharma s.r.l, Rome, Italy). The supplement was delivered orally, with each patient self-administering the tablet on an empty stomach. No group-based administration was used, and all participants received the same dosage. The intervention did not include any incentives for patient participation.

### 4.3. Cervical Cytology

Cervical cytological analysis of HPV-induced lesions was performed using the ThinPrep Papanicolaou Test (Pap test). Cervical cells were collected by inserting the central bristles of the brush and spatula (Cytobrush Plus GT and Pap Perfect Plastic Spatula; CooperSurgical, Trumbull, CT, USA) into the endocervical canal and rotating the brush in a clockwise direction. The spatula containing the scraped cervical cells was then quickly placed into a vial containing 20 mL of PreservCyt^®^ Solution (Cytyc Corp., Marlborough, MA, USA) for cytological analysis. The 2001 Bethesda System for cervical cytology reporting was used to classify the cytological analysis [40].

### 4.4. HPV DNA Test

Detection of HPV DNA was performed using the validated BD OnclarityTM HPV Assay (BD, Franklin Lakes, NJ, USA), following the manufacturer’s instruction. Briefly, the cervical cells (0.5 mL) were collected using a swab and transferred into a BD solution pre-loaded LBC tube to reach a final volume of 2.2 mL. Then, 0.8 mL of viral DNA was sampled and automatically extracted using the BD FOX ™ (BD, Franklin Lakes, NJ, USA) machine. From the final 400 µL of eluted DNA, 50 µL was used for real-time PCR analysis. Detection of the endogenous human beta-globin sequence was used as sample validity control to confirm sample appropriateness, evaluate the efficiency of the extraction process, and ensure amplification performances, as described elsewhere [41,42]. The quality management system implemented at the laboratory is certified according to the reference [43]. 

### 4.5. Statistical Analysis

Statistical analysis was performed using GraphPad Prism software (version 8.0.1, GraphPad Software, San Diego, CA, USA). To compare data at baseline (100% positivity) and after 6 months of treatment, the non-parametric Mann–Whitney U test was used, as the data did not assume a Gaussian distribution. A *p*-value of less than 0.05 was considered statistically significant. All data are presented as mean ± standard error of the mean (SEM).

### 4.6. Safety Evaluation of EGCG: Insights from EFSA Scientific Opinion

In 2018, the European Food Safety Authority (EFSA) conducted a comprehensive safety assessment of EGCG [44]. A prolonged intake (more than 12 months) at a concentration above 866 mg daily may be correlated with hepatoxicity, as indicated by increased levels of transaminases. However, 800 mg EGCG/day is largely outside the range of the daily mean dosage (90 to 300 mg/day) and the daily dose used in this clinical study. Indeed, no side effects were reported at the dosage of 200 mg/day [44].

## 5. Conclusions

HPV management still represents a great challenge in clinical practice due to the absence of effective therapeutic treatments aimed at counteracting infection and its persistence. To date, the prevention and management of HPV infection rely on vaccination and screening programs. Unfortunately, HPV persistence remains an unresolved challenge, and new strategies are needed to address this therapeutic gap.

In this study, we provided further evidence supporting the positive effects of a combination of molecules, including EGCG, FA, B12, and HA, in improving HPV clearance and related cervical lesions, and in counteracting HPV persistence in a large pool of patients compared to previously published studies. Although preliminary ongoing data at 3 months need further analysis to draw definitive conclusions, the positive outcomes obtained after 6 months of treatment corroborate previous evidence in the literature. This study underscores the therapeutic effect of the combination of EGCG, FA, B12, and HA in enhancing HPV clearance and resolving HPV-induced lesions. Furthermore, by counteracting HPV persistence, this approach becomes highly important in reducing exposure of patients to the viral infection and in preventing HPV-associated complications, such as the development of more serious cervical lesions and/or tumoral progression.

## Figures and Tables

**Table 1:** Baseline characteristics of patients enrolled in the study.

Parameters	Treatment Group
Number of patients (*n*)	106
Anatomical sites	Cervical
Age (years)	44.5 ± 8.9
Viral persistence	
- Patients total (*n*)	39
- 1 year	5
- 2 years	20
- 3 years	9
- 4 years	2
- 5 or more years	3
HPV Genotype	
- HR genotype (*n*)	106
- Classification	16, 18, 31, 33, 35, 39, 45, 51, 52, 56, 58, 59, 66, 68
Cervical Lesions	
- Patients positive for lesions (*n*)	39
- Number of lesions	46
- Patient negative for lesions	67
Cytology	
- LSIL/CIN1	36
- ASCUS	5
- HSIL/CIN2/CIN3	5
- Conization	5

## Data Availability

Data are available from the corresponding author upon reasonable request.

## Supplementary Materials

The following supporting information can be downloaded at: https://www.mdpi.com/article/10.3390/ijms26115251/s1.

## References

[1] Schiffman M., Doorbar J., Wentzensen N., de Sanjosé S., Fakhry C., Monk B.J., Stanley M.A., Franceschi S. (2016). Carcinogenic human papillomavirus infection. Nat. Rev. Dis. Primers.

[2] Mlynarczyk-Bonikowska B., Rudnicka L. (2024). HPV Infections-Classification, Pathogenesis, and Potential New Therapies. Int. J. Mol. Sci..

[3] Shanmugasundaram S., You J. (2017). Targeting Persistent Human Papillomavirus Infection. Viruses.

[4] Szymonowicz K.A., Chen J. (2020). Biological and clinical aspects of HPV-related cancers. Cancer Biol. Med..

[5] Koshiol J., Lindsay L., Pimenta J.M., Poole C., Jenkins D., Smith J.S. (2008). Persistent human papillomavirus infection and cervical neoplasia: A systematic review and meta-analysis. Am. J. Epidemiol..

[6] Ciccarese G., Herzum A., Pastorino A., Dezzana M., Casazza S., Mavilia M.G., Copello F., Parodi A., Drago F. (2021). Prevalence of genital HPV infection in STI and healthy populations and risk factors for viral persistence. Eur. J. Clin. Microbiol. Infect. Dis..

[7] de Sanjosé S., Brotons M., Pavón M.A. (2018). The natural history of human papillomavirus infection. Best. Pract. Res. Clin. Obstet. Gynaecol..

[8] Travé G., Zanier K. (2016). HPV-mediated inactivation of tumor suppressor p53. Cell Cycle.

[9] Friedman M.J., Lee H., Kwon Y.C., Oh S. (2022). Dynamics of Viral and Host 3D Genome Structure upon Infection. J. Microbiol. Biotechnol..

[10] Yap J.K.W., Kehoe S.T., Woodman C.B.J., Dawson C.W. (2021). The Major Constituent of Green Tea, Epigallocatechin-3-Gallate (EGCG), Inhibits the Growth of HPV18-Infected Keratinocytes by Stimulating Proteasomal Turnover of the E6 and E7 Oncoproteins. Pathogens.

[11] Wang Y.Q., Lu J.L., Liang Y.R., Li Q.S. (2018). Suppressive Effects of EGCG on Cervical Cancer. Molecules.

[12] Song J.Y., Han J.H., Song Y., Lee J.H., Choi S.Y., Park Y.M. (2021). Epigallocatechin-3-gallate Can Prevent Type 2 Human Papillomavirus E7 from Suppressing Interferon-Stimulated Genes. Int. J. Mol. Sci..

[13] Piyathilake C.J., Henao O.L., Macaluso M., Cornwell P.E., Meleth S., Heimburger D.C., Partridge E.E. (2004). Folate is associated with the natural history of high-risk human papillomaviruses. Cancer Res..

[14] Piyathilake C.J., Badiga S., Paul P., Vijayaraghavan K., Vedantham H., Sudula M., Sowjanya P., Ramakrishna G., Shah K.V., Partridge E.E. (2010). Indian women with higher serum concentrations of folate and vitamin B12 are significantly less likely to be infected with carcinogenic or high-risk (HR) types of human papillomaviruses (HPVs). Int. J. Womens Health.

[15] Jin S., Lin F., Yang L., Zhang Q. (2024). Association between dietary folate intake and HPV infection: NHANES 2005-2016. PLoS ONE.

[16] Qi Z., Ding L., Meng D., Liu H., Wang J., Song L., Lyu Y.J., Jia H.X., Hao M., Tian Z.Q. (2021). [Relationship between serum folate and CIN1 prognosis and its interaction with HR-HPV infection]. Zhonghua Zhong Liu Za Zhi.

[17] Petca A., Borislavschi A., Zvanca M.E., Petca R.C., Sandru F., Dumitrascu M.C. (2020). Non-sexual HPV transmission and role of vaccination for a better future (Review). Exp. Ther. Med..

[18] Woodman C.B., Collins S.I., Young L.S. (2007). The natural history of cervical HPV infection: Unresolved issues. Nat. Rev. Cancer.

[19] Nyman E., Henricson J., Ghafouri B., Anderson C.D., Kratz G. (2019). Hyaluronic Acid Accelerates Re-epithelialization and Alters Protein Expression in a Human Wound Model. Plast. Reconstr. Surg. Glob. Open.

[20] Waggett S., Lyles E., Schlesinger T. (2024). Update on Low-Molecular Weight Hyaluronic Acid in Dermatology: A Scoping Review. EMJ Dermatol..

[21] Yang H., Song L., Zou Y., Sun D., Wang L., Yu Z., Guo J. (2021). Role of Hyaluronic Acids and Potential as Regenerative Biomaterials in Wound Healing. ACS Appl. Bio Mater..

[22] Laganà A.S., Chiantera V., Gerli S., Proietti S., Lepore E., Unfer V., Carugno J., Favilli A. (2023). Preventing Persistence of HPV Infection with Natural Molecules. Pathogens.

[23] Frega A., Gentili C., Proietti S., Lepore E., Unfer V., Fuso A. (2023). Epigallocatechin gallate, folic acid, vitamin B12, and hyaluronic acid significantly increase apoptosis and p53 expression in HeLa cells. Eur. Rev. Med. Pharmacol. Sci..

[24] Aragona C., Bezerra Espinola M.S., Bilotta G., Porcaro G., Calcagno M. (2023). Evaluating the Efficacy of Pervistop((R)), a New Combination Based on EGCG, Folic Acid, Vitamin B12 and Hyaluronic Acid on Patients with Human Papilloma Virus (HPV) Persistent Infections and Cervical Lesions: A Pilot Study. J. Clin. Med..

[25] Tinelli A., Gustapane S., Licchelli M., Coluccia A.C., Panese G., Proietti S., Gambioli R. (2024). Treatment with Epigallocatechin Gallate, Folic Acid, Vitamin B12, and Hyaluronic Acid Decreases HPV Positivity in Women Attending Regional Screening in Puglia. Microorganisms.

[26] Calcagno M., Incocciati B., Di Fraia L., Unfer V. (2024). Counteracting HPV Cervical and Anal Infection through Dietary Supplementation of EGCG, Folic Acid, Vitamin B12 and Hyaluronic Acid: Clinical Case Reports. J. Clin. Med..

[27] Zou C., Liu H., Feugang J.M., Hao Z., Chow H.H., Garcia F. (2010). Green tea compound in chemoprevention of cervical cancer. Int. J. Gynecol. Cancer.

[28] Kciuk M., Alam M., Ali N., Rashid S., Glowacka P., Sundaraj R., Celik I., Yahya E.B., Dubey A., Zerroug E. (2023). Epigallocatechin-3-Gallate Therapeutic Potential in Cancer: Mechanism of Action and Clinical Implications. Molecules.

[29] Xiao S., Tang Y.S., Kusumanchi P., Stabler S.P., Zhang Y., Antony A.C. (2018). Folate Deficiency Facilitates Genomic Integration of Human Papillomavirus Type 16 DNA In Vivo in a Novel Mouse Model for Rapid Oncogenic Transformation of Human Keratinocytes. J. Nutr..

[30] Gao F., Yang C.X., Mo W., Liu Y.W., He Y.Q. (2008). Hyaluronan oligosaccharides are potential stimulators to angiogenesis via RHAMM mediated signal pathway in wound healing. Clin. Investig. Med..

[31] Perkins R.B., Guido R.S., Castle P.E., Chelmow D., Einstein M.H., Garcia F., Huh W.K., Kim J.J., Moscicki A.B., Nayar R. (2020). 2019 ASCCP Risk-Based Management Consensus Guidelines for Abnormal Cervical Cancer Screening Tests and Cancer Precursors. J. Low. Genit. Tract. Dis..

[32] Aggarwal S., Agarwal P., Gupta N. (2024). A comprehensive narrative review of challenges and facilitators in the implementation of various HPV vaccination program worldwide. Cancer Med..

[33] Skinner S.R., Wheeler C.M., Romanowski B., Castellsague X., Lazcano-Ponce E., Del Rosario-Raymundo M.R., Vallejos C., Minkina G., Pereira Da Silva D., McNeil S. (2016). Progression of HPV infection to detectable cervical lesions or clearance in adult women: Analysis of the control arm of the VIVIANE study. Int. J. Cancer.

[34] Grandi G., Botticelli L., Fraia P.D., Babalini C., Masini M., Unfer V. (2023). The Association of Four Natural Molecules-EGCG, Folic Acid, Vitamin B12, and HA-To Counteract HPV Cervical Lesions: A Case Report. J. Pers. Med..

[35] Franco E.L., Villa L.L., Sobrinho J.P., Prado J.M., Rousseau M.C., Desy M., Rohan T.E. (1999). Epidemiology of acquisition and clearance of cervical human papillomavirus infection in women from a high-risk area for cervical cancer. J. Infect. Dis..

[36] Giuliano A.R., Harris R., Sedjo R.L., Baldwin S., Roe D., Papenfuss M.R., Abrahamsen M., Inserra P., Olvera S., Hatch K. (2002). Incidence, prevalence, and clearance of type-specific human papillomavirus infections: The Young Women’s Health Study. J. Infect. Dis..

[37] Brown D.R., Shew M.L., Qadadri B., Neptune N., Vargas M., Tu W., Juliar B.E., Breen T.E., Fortenberry J.D. (2005). A longitudinal study of genital human papillomavirus infection in a cohort of closely followed adolescent women. J. Infect. Dis..

[38] Moscicki A.B., Shiboski S., Broering J., Powell K., Clayton L., Jay N., Darragh T.M., Brescia R., Kanowitz S., Miller S.B. (1998). The natural history of human papillomavirus infection as measured by repeated DNA testing in adolescent and young women. J. Pediatr..

[39] Guo P., Wu L., Wang H., Wang L., Li H., Wang H., Wang Y., Shao S., Chen S. (2025). The Relationship between Systemic Expression Levels of Immune Cells and Tumor Markers and High-Risk HPV Infection in Patients with Cervical Cancer, Cervical Intraepithelial Neoplasia, and Chronic Cervicitis, and its Clinical Significance. Int. J. Womens Health.

[40] Solomon D., Davey D., Kurman R., Moriarty A., O’Connor D., Prey M., Raab S., Sherman M., Wilbur D., Wright T. (2002). The 2001 Bethesda System: Terminology for reporting results of cervical cytology. Jama.

[41] Ejegod D.M., Junge J., Franzmann M., Kirschner B., Bottari F., Sideri M., Sandri M.T., Bonde J. (2016). Clinical and analytical performance of the BD Onclarity™ HPV assay for detection of CIN2+ lesions on SurePath samples. Papillomavirus Res..

[42] Martinelli M., Giubbi C., Sechi I., Bottari F., Iacobone A.D., Musumeci R., Perdoni F., Muresu N., Piana A., Fruscio R. (2022). Evaluation of BD Onclarity™ HPV Assay on Self-Collected Vaginal and First-Void Urine Samples as Compared to Clinician-Collected Cervical Samples: A Pilot Study. Diagnostics.

[43] Enders C., Lang G.E., Lang G.K., Werner J.U. (2017). [ISO 9001:2015 Certification in Quality Management]. Klin. Monbl Augenheilkd..

[44] Younes M., Aggett P., Aguilar F., Crebelli R., Dusemund B., Filipic M., Frutos M.J., Galtier P., Gott D., EFSA Panel on Food Additives and Nutrient Sources added to Food (ANS) (2018). Scientific opinion on the safety of green tea catechins. EFSA J..

