# Supplementary material for: Oral Treatment with EGCG, Folic Acid, Vitamin B12, and Hyaluronic Acid Improves HPV Clearance and Counteracts Its Persistence: A Clinical Study
Source: Int J Mol Sci. 2025 May 29;26(11):5251. doi: 10.3390/ijms26115251 (PMC12155352; doi:10.3390/ijms26115251)
Supplement: Supplementary file 1 [file ijms-26-05251-s001.zip › ijms-3617516-supplementary.pdf]

7. Supplementary data

7.1 Supplementary materials and methods

7.1.1 Baseline characteristics of patients undergoing to 3-month-treatment regimen

A total of 21 patients, with a mean age of 47.5 years and meeting the selection criteria outlined in the "Materials and Methods" section (paragraph 2.1), were evaluated after 3 months of treatment. Among the selected patients, 5 presented low grade HPV-induced cervical lesions confirmed by cytological analysis of ThinPrep Pap Test, and they were further stratified according to cytological classification into LSIL/CIN1 (n=4) and ASCUS (n=1).
All methods, including the treatment regimen, cervical cytology, HPV DNA testing, and statistical analysis, were conducted as previously described in the "Materials and Methods" section.

7.1.2 Comparison of HPV clearance rate with spontaneous regression of previously published studies

A detailed comparison of HPV clearance rates was carried out with previously published studies on spontaneous HPV clearance. Clinical studies [36-39], with varied demographic characteristics (ethnicity, number of patients and age range) were selected for comprehensive analysis. In addition, control patients from previously published studies [21,22] were included to provide a stronger basis for comparison. Data extrapolation at selected time points, baseline ("0 months"), 3 and 6 months, was performed using WebPlotDigitizer tool (https://automeris.io/docs/). Kaplan-Meier curves of selected studies were generated and compared with treatment HPV clearance rates.

7.2 Supplementary results

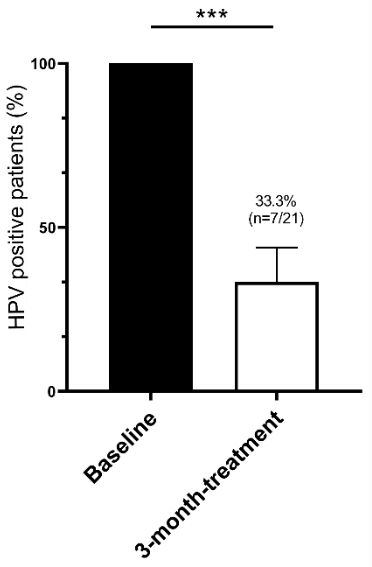

Supplementary Figure S1. The effect of 3-month treatment with EGCG, FA, B12, and HA on HPV clearance. Graphical representation of the effect of EGCG, FA, B12, and HA on HPV clearance after 3 months of treatment. At the baseline (black column), all patients were HPV-positive (100%). After 3 months of treatment (white column), 66.7% (14 out of 21 patients) achieved HPV clearance, while 33.3% (7 out of 21 patients) remained HPV-positive. Data are reported as means ± standard error mean. Statistical significance was evaluated using the Mann-Whitney U test for non-parametric comparison; * $p < 0.05$, ** $p < 0.01$, or *** $p < 0.001$.

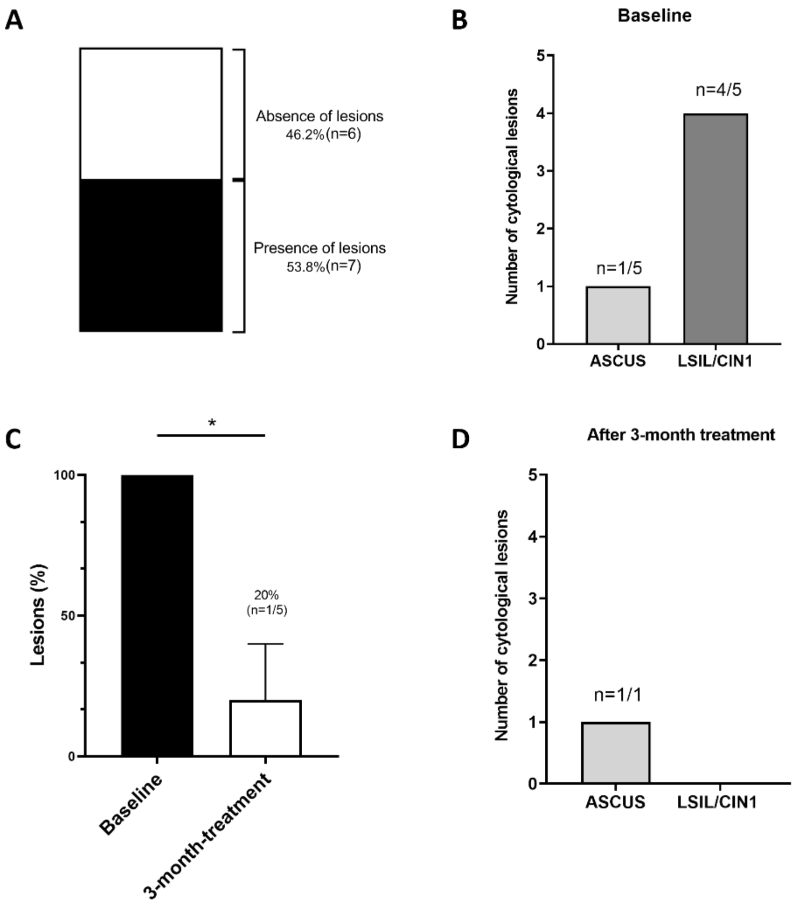

Supplementary Figure S2. The effect of 3-month treatment with EGCG, FA, B12, and HA on HPV-induced cervical lesions. Baseline distribution of cervical lesions in HPV-positive patients underwent a 3-month treatment (A) and stratified by cytological classification (B). Graphical representation of the effect of EGCG, FA, B12, and HA on HPV-induced cervical lesions (C), stratified by cytological classification (D). All patients included in the statistical analysis initially presented HPV-induced cervical lesions, with the baseline condition (black column) of lesions' presence set at 100% (C). After 3 months of treatment (white column), 80% (4 out of 5 patients) resolved HPV-induced lesions, while 20% (1 out of 5 patients) continued to exhibit unresolved lesions. Data are reported as means ± standard error mean. Statistical significance was evaluated using the Mann-Whitney U test for non-parametric comparison; * $p < 0.05$, ** $p < 0.01$, or *** $p < 0.001$. Abbreviations: ASCUS (Atypical squamous cells of undetermined significance); LSIL (Low-grade squamous intraepithelial lesion) and CIN1 (Cervical intraepithelial neoplasia grade 1).

A

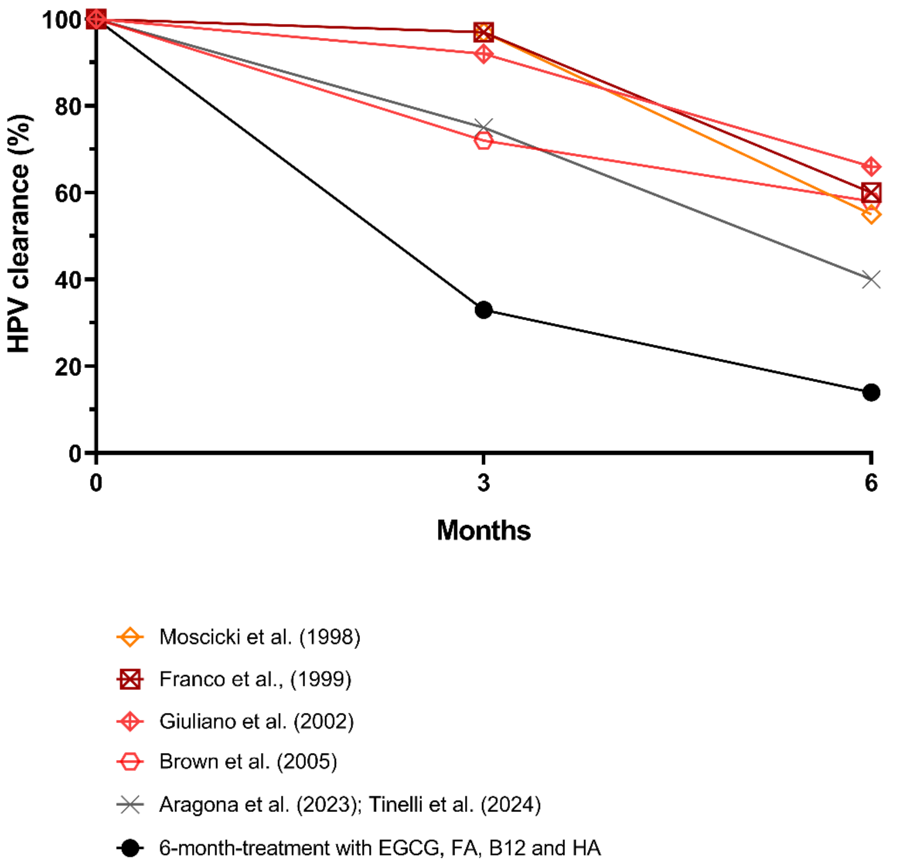

B

Months	Moscicki et al. (1998)	Franco et al., (1999)	Giuliano et al. (2002)	Brown et al. (2005)	Aragona et al. 2023; Tinelli et al. 2024	6-month treatment with EGCG, FA, B12 and HA
0	100	100	100	100	100	100
3	97	97	92	72	75	33
6	55	60	66	58	40	14

Supplementary Figure S3. Comparison of HPV clearance at 3 and 6 months with spontaneous regression data reported in previous studies. Comparison of HPV clearance rate at 3 and 6 months after 6-month-treatment, with spontaneous regression of previously published studies (i.e., Giuliano et al. (2002); Franco et al. (1999); Moscicki et al. (1998); Brown et al. (2005)). Additionally, spontaneous regression data from both previously published studies and raw data (i.e., Aragona et al., 2023; Tinelli et al., 2024), were included as further controls in the comparison considering their recent release. (A) 6-month-treatment with EGCG, FA, B12 and HA highlights trends toward HPV clearance at both investigated timepoints (66.7% at 3 months and 85.8% at 6 months) in comparison with previously published studies. (B) Table summarizing the mean percentage of HPV positivity at defined time points (i.e., 0, 3 and 6 months). Data extrapolation for superposition curves at 3- and 6-months' timeframes was performed using WebPlotDigitizer tool (https://automeris.io/docs/).
